# Establishment and characterization of novel epithelial-like cell lines derived from human periodontal ligament tissue in vitro

**DOI:** 10.1007/s13577-017-0173-y

**Published:** 2017-04-22

**Authors:** Kallapat Tansriratanawong, Hiroshi Ishikawa, Junko Toyomura, Soh Sato

**Affiliations:** 1 Department of Oral Medicine and Periodontology, Faculty of Dentistry, Mahidol University, 6 Yothi Street Rajthevi, Bangkok, 10400 Thailand; 2 Department of NDU Life Sciences, Nippon Dental University, Tokyo, Japan; 3 Department of Periodontology, Nippon Dental University, Niigata, Japan

**Keywords:** Epithelial-like cells, Establishment cell lines, Periodontal ligament tissue, Epithelial rests of Malassez differentiation, EMT

## Abstract

In this study, novel human-derived epithelial-like cells (hEPLCs) lines were established from periodontal ligament (PDL) tissues, which were composed of a variety of cell types and exhibited complex cellular activities. To elucidate the putative features distinguishing these from epithelial rest of Malassez (ERM), we characterized hEPLCs based on cell lineage markers and tight junction protein expression. The aim of this study was, therefore, to establish and characterize hEPLCs lines from PDL tissues. The hEPLCs were isolated from PDL of third molar teeth. Cellular morphology and cell organelles were observed thoroughly. The characteristics of epithelial–endothelial-mesenchymal-like cells were compared in several markers by gene expression and immunofluorescence, to ERM and human umbilical-vein endothelial cells (HUVECs). The resistance between cellular junctions was assessed by transepithelial electron resistance, and inflammatory cytokines were detected by ELISA after infecting hEPLCs with periodontopathic bacteria. The hEPLCs developed into small epithelial-like cells in pavement appearance similar to ERM. However, gene expression patterns and immunofluorescence results were different from ERM and HUVECs, especially in tight junction markers (Claudin, ZO-1, and Occludins), and endothelial markers (vWF, CD34). The transepithelial electron resistance indicated higher resistance in hEPLCs, as compared to ERM. Periodontopathic bacteria were phagocytosed with upregulation of inflammatory cytokine secretion within 24 h. In conclusion, hEPLCs that were derived using the single cell isolation method formed tight multilayers colonies, as well as strongly expressed tight junction markers in gene expression and immunofluorescence. Novel hEPLCs lines exhibited differently from ERM, which might provide some specific functions such as metabolic exchange and defense mechanism against bacterial invasion in periodontal tissue.

**Electronic supplementary material:**

The online version of this article (doi:10.1007/s13577-017-0173-y) contains supplementary material, which is available to authorized users.

## Introduction

Periodontal ligament (PDL) tissue is a specialized and complex connective tissue of ectomesenchymal origin. PDL attaches tooth root cementum to alveolar bone for reciprocal support of the periodontium and tooth. PDL plays a pivotal roles in homeostasis, maintenance, and regeneration of periodontium. The heterogeneous cell population in PDL tissue is comprised of various cell types including fibroblasts, endothelial cells, epithelial cells, PDL progenitor cells, and inflammatory cells. The differentiation capacity and differentiation stage of the cell populations of PDL tissue depend on the expedient environment [1–4]. Therefore, the PDL cell populations and their functions have not yet been completely characterized.

Root development involves complicated cell interactions and signal transductions. Dental epithelial cells, which line the inner and outer layers, proliferate to form double layers, known as Hertwig’s epithelial root sheath (HR), extending longitudinally from the cervical loop to apical foramen. HR create networks of epithelial cells, which then disintegrate into cell clusters referred to as “epithelial rests of Malassez” (ERM) after the roots form [5–7]. ERM appear in clusters of polyhedral-shaped epithelial cells with a high nuclear-cytoplasmic ratio, which are thought to be involved in maintaining the PDL space, cellular activity, paracrine secretion of cytokines or growth factors, and wound healing of PDL tissue [2]. Their actual function and differentiated characters; however, remain unclear. Studies of isolated ERM have revealed unique characteristics, which are thought to be involved in the regeneration of the PDL attachment apparatus and in PDL repair [8–12]. Moreover, ERM cells may be involved in the repair and regeneration of cementum, which requires an epithelial–mesenchymal transition and differentiation into cementoblasts, and acellular cementum formation during the initial stage of root formation [13–15]. ERM are presumed to be terminal-stage epithelial cells, and thus ERM functions may be limited. ERM have many characteristics according to their water and ground substance content, and differentiate into several different cell types. The contribution of ERM to PDL tissue may be to facilitate ion or water transport, especially by specific junctional organelles, including gap junctions, desmosomes, hemidesmosomes, and tight junctions (TJ). How ERM contribute to defense mechanisms to eliminate periodontopathic bacterial invasion; however, remains unclear.

Our laboratory routinely isolates ERM cells from PDL tissues, which have features such as polyhedral epithelial-like differentials among the predominant fibroblast-like cells in PDL tissue (data not shown). Cultivation of ERM cells in vitro provides the opportunity to study features such as polyhedral shape, and high nuclear-cytoplasmic ratio in monolayers. Upon prolonged culturing; however, we were surprised to discover that some isolated epithelial-like cells produced distinct spherical colonies formation in multilayers. We hypothesized that these epithelial-like cells might differentiate from ERM, or possibly from uncommitted progenitor cells among ERM or periodontium, thereby providing novel insight into the functions of PDL tissues.

Therefore, we sought out to understand the cellular activities of novel epithelial-like cell lines derived from PDL tissues and to clarify their functional involvement. The objective of this study was to establish and characterize novel human-derived epithelial-like cells (hEPLCs) lines from PDL tissue.

## Materials and methods

### Cell culture

All subjects who participated in this study provided written informed consent. The protocol was approved by the ethics committee of Nippon Dental University. Human fully or partially erupted third molar teeth were obtained from five healthy female Japanese subjects (20–28-years-old) by surgical extraction. PDL tissues were scraped from the middle third of the root surface using sterile surgical blades after sterilizing the surface with iodine. The tissue was washed with Hanks’ Balanced Salt Solution (HBSS; Nissui Pharmaceutical Co., Ltd., Tokyo, Japan) and cut into small pieces. The pieces were digested with 0.1 mg/mL collagenase type I (Sigma-Aldrich, St. Louis, MO, USA) and 0.4 mg/mL dispase (Wako Pure Chemical Co. Ltd., Tokyo, Japan) for 1 h (h) at 37 °C. The suspensions were filtered through 70-μm cell strainers (Falcon, BD Labware, Franklin Lakes, NJ, USA) and then centrifuged at 300×*g* for 5 min, and the retrieved cells were further cultured in growth medium (GM). GM contained Dulbecco’s modified Eagle’s medium/Ham’s nutrient mixture F12 (DMEM/F12; Gibco BRL, Carlsbad, CA, USa) supplemented with 15% fetal bovine serum (FBS; Lot No. 12E109, Sigma-Aldrich), 2 mM glutamine (GlutaMAX I, Invitrogen, Carlsbad, CA, USA), 50 U/mL penicillin, 50 μg/mL streptomycin (Gibco BRL), and 0.25 µg/mL Fungizone (Gibco BRL). After 2–3 days, adherent cells from primary PDL cell culture were demonstrated in heterogeneous, comprising mostly in fibroblast-like cells combined with some endothelial- and epithelial-like cell morphologies. We particularly picked up single cell which expressed in the epithelial-like cell morphology among these heterogeneous primary cell cultures using small paper filter soaked with digestive solution, 0.1% trypsin (Becton-Dickinson, Franklin Lakes, NJ, USA) and 0.02% ethylenediaminetetraacetic acid (EDTA; Dojindo, Kumamoto, Japan) in phosphate-buffered saline without calcium and magnesium (PBS[−]). This single cell isolation was a modified method from classical colony isolation by cloning ring technique [16]. The clonal cells were then isolated into 24-well plates (Nunc, Thermo Fisher Scientific, Pittsburgh, USA) and expanding to 60-mm^2^ culture dishes (Nunc) after reached to 70–80% confluence in the well under 37 °C/4.7% CO_2_ conditions. When cells reached more than 70–80% confluence in 60-mm^2^ culture dishes, they were split into a 1:3 dilution using 0.1% trypsin and 0.02% EDTA/PBS[−], and divided for cryopreservation at −80 °C until further use. The derived epithelial-like cells, which further grew into multilayers forming spheroidal structure were established and named as hEPLCs (human-derived epithelial-like cells). The ERM cell line, which was established in our laboratory with similar method of hEPLCs and a commercial human umbilical-vein endothelial cells (HUVECs; Lonza Walkersville, Walkersville, MD, USA) lines were used for comparison with hEPLCs.

### Cell morphology

hEPLCs from passage 1st, which were sub-cultured from primary culture by 0.1% trypsin and 0.02% EDTA, were then isolated and re-seeded at a concentration of 10^3^ cells/cm^2^ in 60-mm^2^ culture dishes. Their growth was evaluated from day 1 through 21 using a phase-contrast microscope. ERM and HUVECs were used for comparison of the morphologies at a confluence stage.

### RNA extraction and reverse transcriptase PCR (RT-PCR)

Total RNA of hEPLCs, HUVECs, and ERM cells from 60-mm^2^ culture dishes were extracted using the RNeasy Mini kit (Qiagen, Hilden, Germany) according to the manufacturer’s protocol and the quantity of RNA was determined by 260/280 nm absorbance. Complementary DNA (cDNA) was synthesized from 1-µg RNA using the High Capacity cDNA Synthesis kit (Applied Biosystems, Carlsbad, CA, USA). The PCR Supermix Platinum kit (Invitrogen) was used, followed by preincubation at 94 °C for 2 min, 35 cycles of denaturation at 94 °C for 30 s, primer annealing (Supplementary Table 1) for 30 s, and extension at 72 °C for 1 min. Finally, we performed a post-extension step at 72 °C for 7 min. PCR products were electrophoresed using 2% agarose gel at 100 mA for 35 min before being stained with 0.5 µg/mL ethidium bromide. Primers were designed for evaluated mRNA expression, including Claudin-1, Claudin-2, Claudin-3, Zonula Occludens 1 (ZO-1 or TJP-1), Occludins (OCLN), CD34, Amelogenin, Ameloblastin, von Willebrand factor (vWF) as shown in Supplementary Table 1. Glyceraldehyde 3-phosphate dehydrogenase (GAPDH) was used as an endogenous control.

### Three-dimensional (3D) cell pellet culture and immunofluorescence

For investigation lineage specificity and epithelial cell characteristics, hEPLCs were cultured in 3D technique using cells concentration of 10^7^ cells/mL in a 15-mL filter top tube (CELLSTAR^®^, Greiner bio-one). Then, cells were centrifuged at 300×*g* for 15 min to obtain precipitated pellets at the bottom of the tubes under culture conditions at 37 °C/4.7% CO_2_. After cultured for 2 week, the 3D-cell pellet cultures, which were formed detachable spheroid pellet at the bottom of the tubes, were then fixed with 10% formalin for 10 min. Formalin was removed and then pellets were replaced with 50% ethanol for 30 min, 70% ethanol for 30 min, 90% ethanol for 30 min, 95% ethanol for 30 min, and 100% ethanol twice for 30 min, respectively. Half of the 100% ethanol was removed and replaced with xylene for 30 min. Pellets were washed twice in 100% xylene for 30 min each and were embedded in paraffin followed by serial sectioning at 5 µm. The sections were de-paraffined and re-hydrated with three washes (5 min each) in 100% xylene and three washes in a descending series of ethanol (100, 95, and 90%). For immunofluorescence, the 3D-cells were washed three times with PBS[−], and then fixed with ice cold methanol (Wako Pure Chemical) for 10 min at 4 °C. After washing three times with PBS[−], 1% bovine serum albumin/PBS[−] was used to block non-specific protein interactions for 30 min at room temperature. Then, primary antibodies were used as follows: monoclonal mouse anti-cytokeratin-14 (CK-14, 1:400, Millipore), monoclonal mouse anti-human mitochondria (1:200, Millipore), monoclonal mouse anti-vimentin (1:200, Sigma-Aldrich), polyclonal rabbit anti-amelogenin (1:200, Santa Cruz), polyclonal rabbit anti-ameloblastin (1:200, Abcam), polyclonal rabbit anti-claudin-1 (1:1000, Sigma-Aldrich), and polyclonal rabbit anti-ZO-1 (1:1000, Sigma-Aldrich), polyclonal rabbit anti-OCLN (1:1000, Sigma-Aldrich), and polyclonal rabbit anti-von Willebrand factor (vWF, 1:2000, Sigma-Aldrich) were added and the cells were incubated overnight at 4 °C. Rabbit-derived Alexa Fluor 488 or mouse-derived Alexa Fluor 568-conjugated secondary antibodies were diluted at 1:500 and incubated for 30 min. The cells were then stained with 4′, 6-diamine-2-phenylindol. Primary antibody was omitted as a negative control. All images were captured using a fluorescence microscope (BZ-9000, KEYENCE, Tokyo, Japan).

### Transepithelial electrical resistance (TEER)

hEPLCs and ERM were retrieved from culture dishes for the TEER assay. A 1-mL hEPLCs and ERM suspension was seeded in 6-well plates (Nunc) with a cell concentration of 10^4^ cells/mL. When cells reached to 7–10 days, TEER measurements and paracellular permeability provide information regarding cellular barrier properties were analyzed by measuring hEPLCs with a volt-ohm meter using the STX-2 electrode system (Millipore, Schwalbach, Germany).

### Transmission electron microscope (TEM)

We used TEM for investigate cellular organelles of hEPLCs and bacterial interactions of hEPLCs infection. hEPLCs were cultured for 7–10 days in 35-mm^2^ culture dishes. The cells were fixed using 2.5% glutaraldehyde in 0.1 M PBS[−] for 1 h at room temperature, and then post-fixed with 1% OSO_4_ at 0 °C for 30 min. The cells were rinsed with buffer, dehydrated with ethanol, immersed twice in absolute propylene oxide, and embedded in Quetol 812 prior to evaluating their morphology. Sections were cut at a thickness of 90–100 nm with a diamond knife and mounted onto grids. Following staining with uranyl acetate and lead citrate, the sections were observed using a JEOL JEM-1200 EX-II electron microscope at 80 kV.

### Bacterial strains and culture conditions

The periodontopathic bacteria, including *Porphyromonas gingivalis* (*Pg*) strain W83 and 33277 and *Aggregatibacter actinomycetemcomitans* (*Aa*), were maintained under anaerobic conditions using the GasPak™ EZ Anaerobe Pouch System (BD Bioscience) at 37 °C. Blood agar plates supplemented with 5 µg/mL hemin (Sigma-Aldrich), 0.5 µg/mL menadione (Sigma-Aldrich), and tryptic soy selective agar supplemented with 10% horse serum and 1 g/L yeast extract were used as the bacterial culture medium for *Pg* and *Aa* [17].

### Infection of hEPLCs with bacteria

hEPLCs were inoculated with *Pg* or *Aa* and incubated for 30 min, 2, 4, and 24 h. The cells were washed twice with PBS[−] and the bacterial concentration was then determined by optical density at 600 nm (OD_600_) using a path length of 0.5 mm. The resulting colonies had approximately 5 × 10^8^ colony-forming unit per milliliter for using as bacterial stocks. The stocks of bacterial suspension were further diluted in cell culture medium without antibiotics for all infection experiments. The preparation of bacterial method was modified from Gründler et al. [18].

### Determination of inflammatory cytokines by enzyme-linked immunosorbent assay (ELISA)

The supernatant of hEPLCs infected with bacteria collected from each time interval at 30 min, 2, 4, and 24 h was analyzed by ELISA following the manufacturer’s instructions. Briefly, each 50 µL of assay diluent and the supernatants were incubated in 96-well plates at 37 °C for 2 h. The plates were washed four times. conjugated to each cytokines (R&D Systems, RayBio^®^, Tokyo, Japan) of prostaglandin E2 (PGE2), interleukin-8 (IL-8), and tumor necrosis factor alpha (TNF-α) were added to each plate and the plates incubated further for 2 h. After washing, 100 µL of peroxidase substrate solution was added and the plates were incubated for 30 min and the reaction was stopped. Optical density was determined within 30 min using an ELISA microplate reader at 450 nm.

### Karyotype analysis

Karyotype was analyzed in sub-confluent cells after incubation with 100 nM colcemid (Sigma-Aldrich) for 4 h at 37 °C. The cells (10^4^ cells/mL) were then retrieved, centrifuged, and re-suspended in 70 mM KCl for 20 min at 37 °C. The cells were then fixed with a methanol:acetic acid (3:1) solution for 5 min at room temperature and repeatedly centrifuged. The cell pellets were re-suspended in methanol:acetic acid and incubated overnight at 4 °C. Aliquots of cell suspension were dropped onto a cold micro-glass slide followed by staining with Giemsa solution. Fifty mitotic figures were randomly selected, and chromosomal distribution was counted to determine the karyotype.

### Statistical analysis

Data of TEER analysis are reported as mean ± standard deviation using unpaired Student’s *t* tests for two-group comparisons. Repeated measure ANOVA was used to analyze the cytokine secretion experiments. Statistical analysis was performed using SPSS 18 for Windows. Experiments were performed in triplicate. A *P* value of <0.05 was considered statistically significant.

## Results

### Cell morphologies in phase-contrast micrograph

**Fig. 1:**
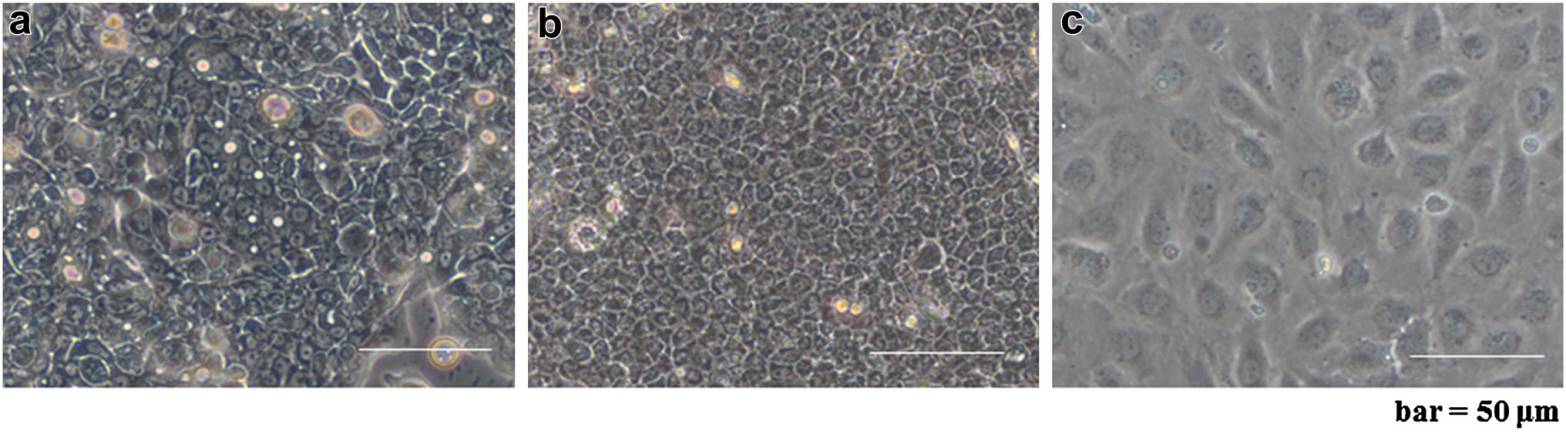
Morphologic characterizations of **a** human-derived epithelial-like cells (hEPLCs), **b** epithelial rests of Malassez (ERM), and **c** umbilical-vein endothelial cells (HUVECs) in phase-contrast microscope. hEPLCs and ERM appearances demonstrated highly orientated in pavement cellular arrangement with evenly small whereas HUVECs had a cobblestone appearance with large dark nuclei. *Scale bar* 50 µm

Comparison of cell morphologies using phase-contrast microscope indicated the hEPLCs, ERM, and HUVECs, which were demonstrated in Fig. 1a–c, respectively. Pavement-like arrangement was observed in monolayered hEPLCs similar to ERM. The hEPLCs and ERM were highly orientated and presented in an evenly small. On the contrary, HUVECs were identified in cobblestone appearance with large dark nuclei, and high nuclear-cytoplasmic ratio. Although, ERM cell morphology was similar to that of the hEPLCs, the ERM grew in a monolayer after reaching to the confluence (Fig. 1b) whereas hEPLCs grew in spherical appearance after 3-weeks culturing (Supplementary Fig. 1). The Cell morphologies of hEPLCs were sequentially observed from day 1 to day 21 under a phase-contrast microscope. The morphology features demonstrated polyhedral and oval shapes from day 1 to day 3 (Supplementary Fig. 1A and 1B). The hEPLCs growth divided and expanded from the center of cell–cell attachment speculating that formed tight contact with each other through their nuclear membranes. Cells from day 7 developed a homogenous polyhedral epithelial-like cell morphology (Supplementary Fig. 1C), and from day 7 to day 10, hEPLCs developed in monolayer appearance (Supplementary Fig. 1C and 1D). At 2 weeks, the hEPLCs colonies became dense in cellular aggregation and started to develop in multilayers appearance (Supplementary Fig. 1E). The colonies gradually aggregated in tight spherical colonies over the next 3 weeks (Supplementary Fig. 1F).

### Gene expression of hEPLCs

**Fig. 2:**
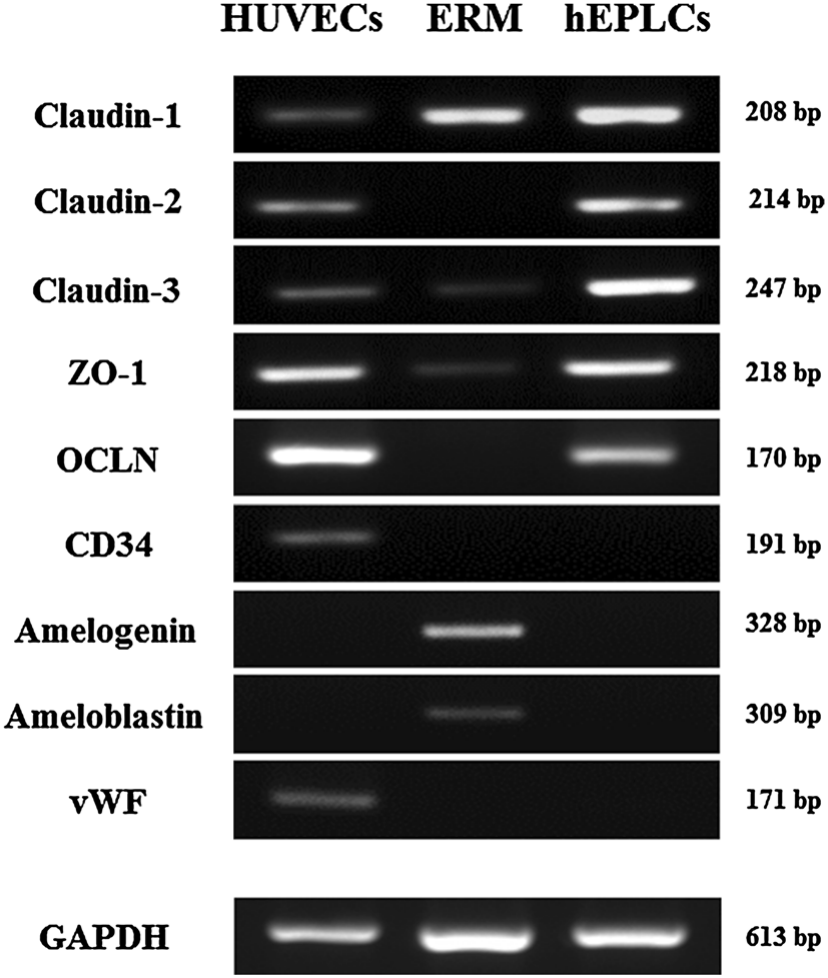
Reverse transcriptase PCR (RT-PCR) were used to identify mRNA expression of TJ genes; Claudin-1, Claudin-2, Claudin-3, Zonula Occludens 1 (ZO-1), and Occludins (OCLN) among hEPLCs. HUVECs and ERM were used for comparison in lineage specifications. GAPDH was used as an endogenous control

RT-PCR (Fig. 2) results confirmed the mRNA expression of markers among HUVECs, ERM, and hEPLCs. The hEPLCs strongly expressed in all TJ markers, including Claudin-1, Claudin-2, Claudin-3, ZO-1, and OCLN similarly to HUVECs whereas ERM showed mRNA expression of Claudin-1, and weakly expressed of Claudin-3, and ZO-1. Unlike HUVECs, hEPLCs and ERM negatively expressed CD34, which is a potent hematopoietic lineage marker and endothelial marker, vWF. Moreover, amelogenin and ameloblastin, which particularly detected in inner and outer enamel epithelial cells, were expressed in ERM contrast to hEPLCs.

### Characterizations of hEPLCs in immunofluorescence of TJ proteins, epithelial adhesion molecules, and endothelial markers

**Fig. 3:**
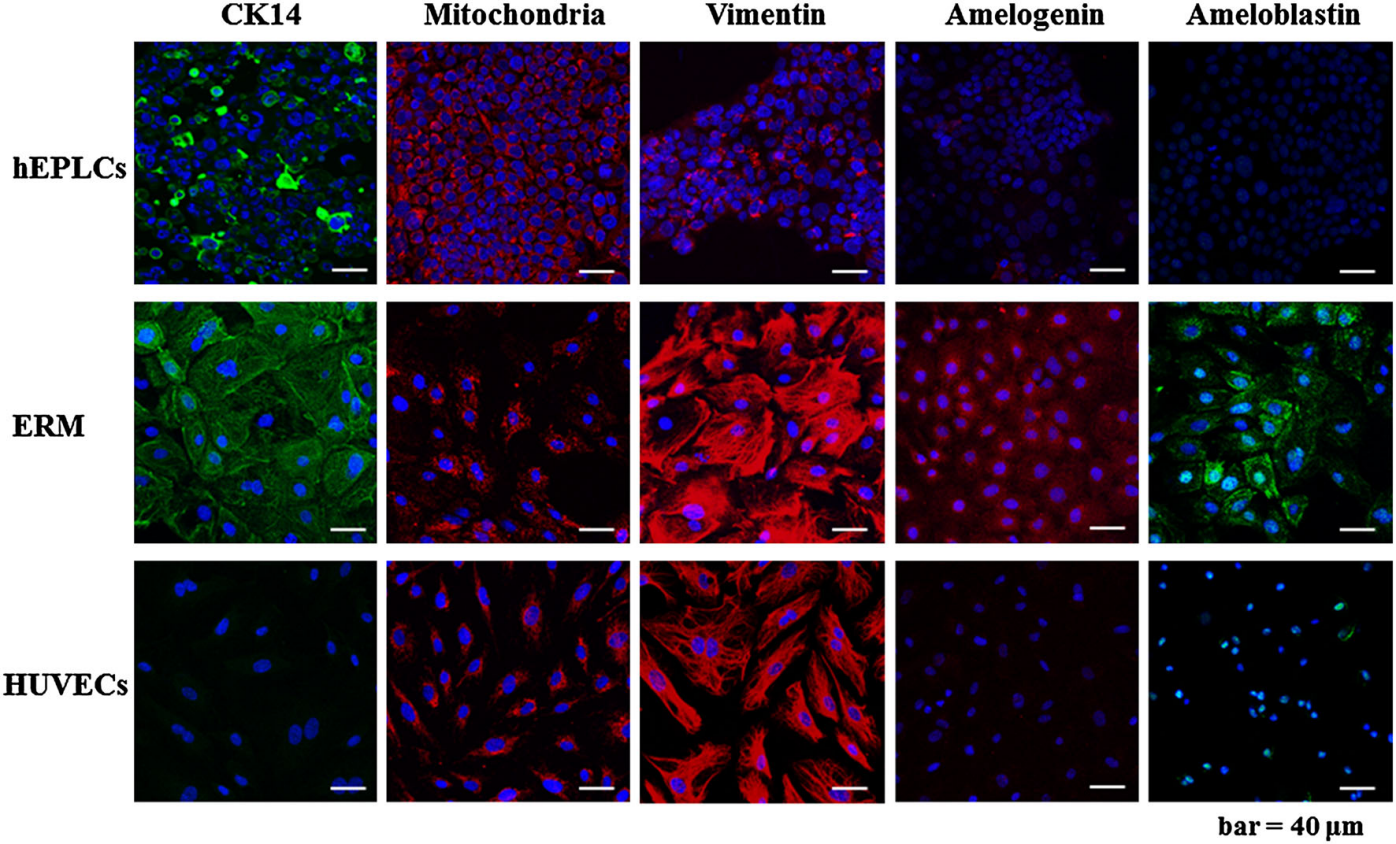
Immunofluorescence studies indicated the expression of epithelial cell markers; cytokeratin-14 (CK14), mesenchymal markers; mitochondria and vimentin in hEPLCs, but negatively expressed in amelogenin and ameloblastin. *Scale bar* 50 µm

**Fig. 4:**
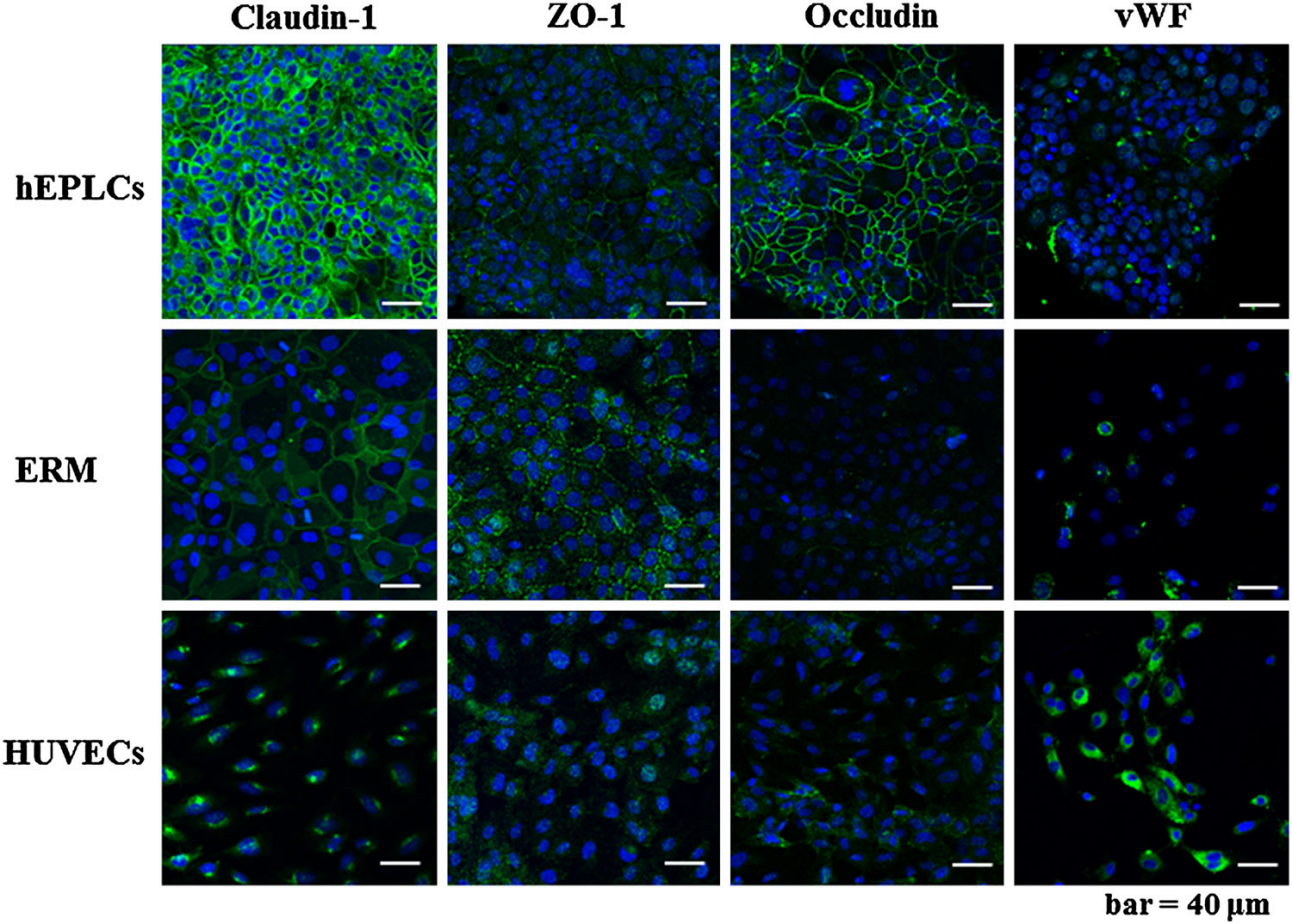
Immunofluorescence studies indicated the strong expression of the TJ proteins Claudin-1, Zonula Occludens 1 (ZO-1), and Occludins (OCLN) markers. The hEPLCs and ERM were negative for detection of endothelial cell marker; von Willebrand factor (vWF). *Scale bar* 50 µm

Slide sections of monolayered hEPLCs were de-paraffined and then immunohistochemically stained to detect the expression of various markers. The hEPLCs and ERM were expressed of CK-14 protein in their cytoplasm, which is a principal odontogenic epithelium locating in almost all cells of enamel organ. However, hEPLCs were partially positive stained when compared to ERM (Fig. 3). Mitochondria and vimentin markers were used as a positive control of cells derived from mesenchymal lineage showing strongly positive stained HUVECs, ERM, and hEPLCs (Fig. 3). Interestingly, hEPLCs were negatively stained for both Amelogenin and Ameloblastin markers contrasting to ERM (Fig. 3). For TJ markers, hEPLCs strongly demonstrated expression in all markers together with Claudin-1, ZO-1, and OCLN (Fig. 4). Positive staining was detected at the plasma membrane domain of paracellular area in cell–cell adhesion as similar to ERM (Claudin-1 and ZO-1) whereas HUVECs had moderately showed in cytoplasm of Claudin-1, ZO-1, and OCLN marker staining. However, ERM could not be detected completely in OCLN marker. For endothelial marker; vWF, ERM and hEPLCs revealed very weak staining of this marker contrasting to HUVECs.

### hEPLCs exhibited high cellular resistance of cell–cell adhesion

**Fig. 5:**
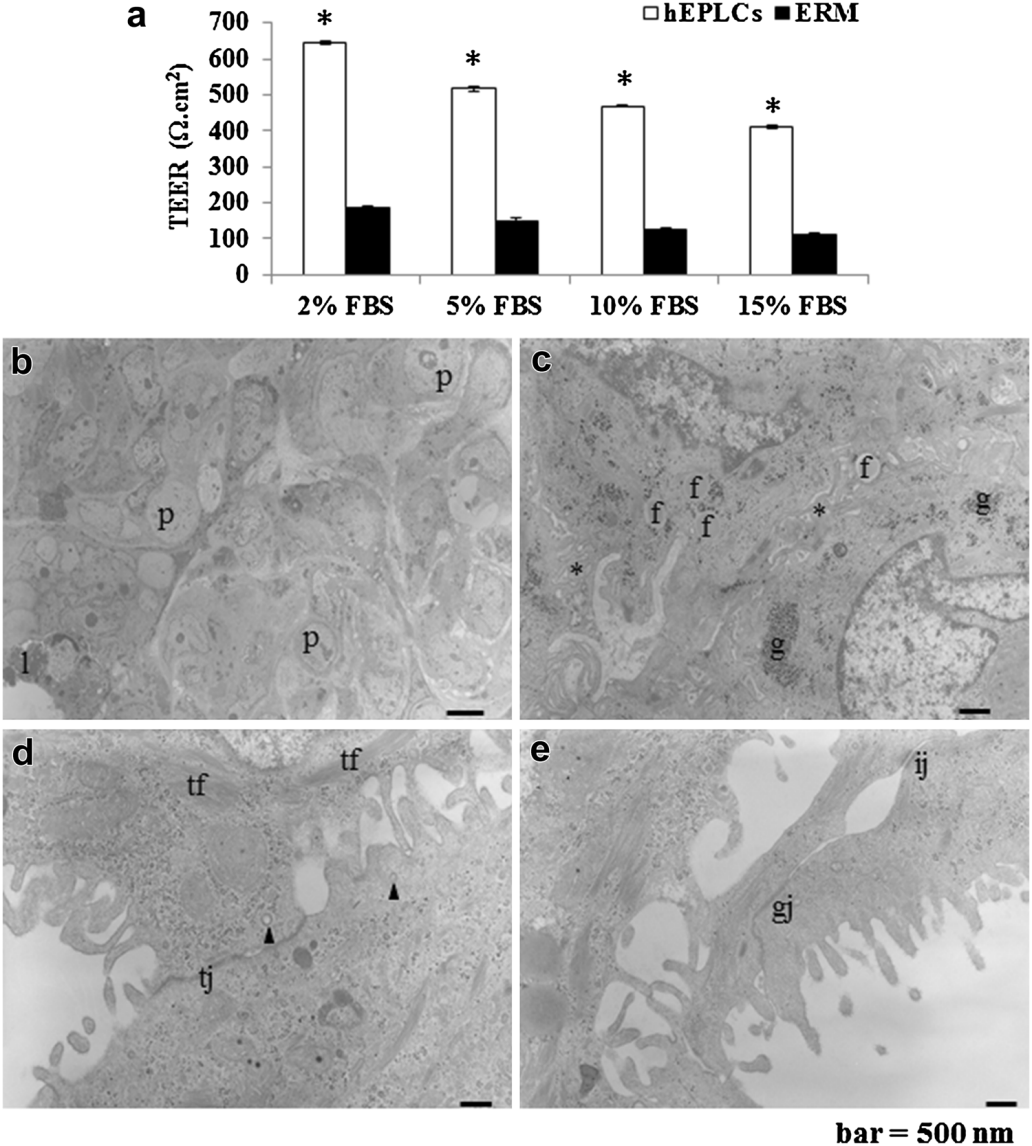
Transepithelial electrical resistance was significantly higher, indicating that hEPLCs have higher resistance than epithelial rests of Malassez (ERM) at all FBS concentrations (**a**, *P < 0.05, *n* = 5). **b** Representative micrographs of transmission electron microscopy of hEPLCs organelles were depicted as follows; phagosomes (p) and lysosomes (l) were often observed in the cytoplasm. **c** Interdigitations with microvilli caused clusters to form within the intercellular space (*asterisks*). Fat droplets (f) and glycogen granules (g) were abundant. **d** Illustration demonstrates the long TJ (tj) contact between intercellular junctions. Tonofilaments (tf) were relatively well developed in the cytoplasms. Coated vesicles (*arrowheads*) were often observed. **e** Intermediate junctions (ij) and gap junctions (gj) are shown. *Scale bar* 500 nm

TEER indicated a high resistance between cell-to-cell barriers of hEPLCs from monolayer cell culture higher than ERM when compared in all FBS concentrations examinations (*P* < 0.05, Fig. 5a), including 2% FBS (hEPLCs; 643.67 ± 4.5 and ERM; 185.33 ± 7.14), 5% FBS (hEPLCs; 517.83 ± 7.28 and ERM; 149.83 ± 7.19), 10% FBS (hEPLCs; 469.33 ± 4.13 and ERM; 126.5 ± 2.74), and 15% FBS (hEPLCs; 411.83 ± 4.27 and ERM; 112 ± 4), respectively. Most of the specialized organelles were detected in the hEPLCs by TEM (Fig. 5b–e). Large phagosomes (p) and lysosomes (l) were observed in the cytoplasm (Fig. 5b). All cells had a high nucleus to cytoplasm ratio, indicating their high active functions, such as killing invading microorganisms. Moreover, the nucleus morphology was irregular, either round or oval, with indentations, suggesting the potential digestive function of hEPLCs. These cells also had interdigitations with microvilli in (asterisks, Fig. 5c), suggesting that they could lock together, thereby, enhancing their mechanical and chemical functions to destroy microorganisms. Fat droplets (f) and glycogen granules (g) were generally observed (Fig. 5c). hEPLCs had long TJ (tj) at cell–cell interactions (Fig. 5d). Moreover, bundles of tonofilaments (tf) were generally observed. The hEPLCs also had the characteristics of secretory cells, such as coated vesicles (arrowheads, Fig. 5d). In addition to TJ, we also detected intermediate junctions (ij) and gap junctions (gj) (Fig. 5e). These structures might enhance the selective penetration of several substances into the cells.

### Inflammatory cytokine secretion after bacterial inoculation

**Fig. 6:**
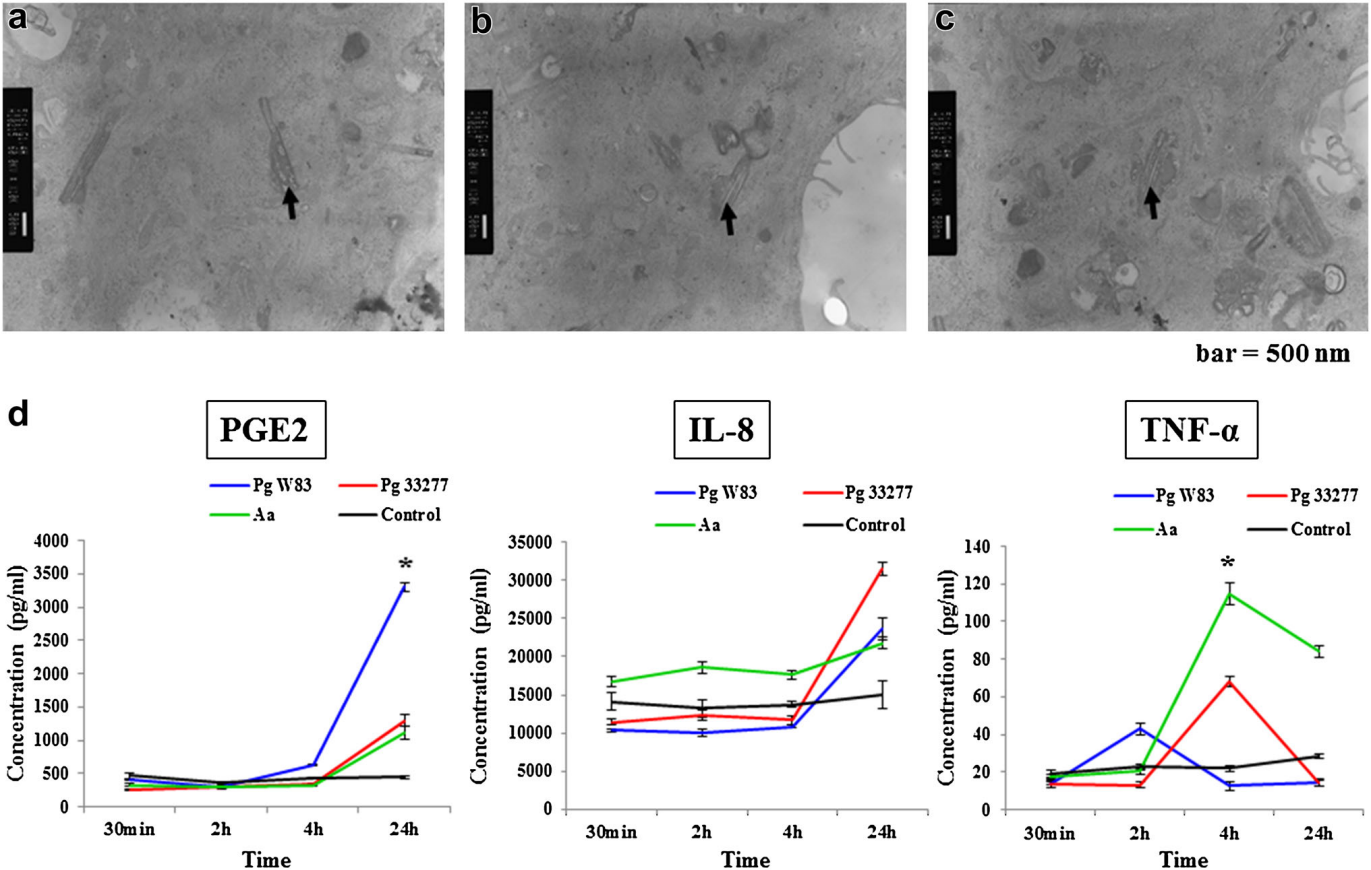
Representative transmission electron microscopy micrographs showing bacterial phagocytosis at 2 h (**a**), 4 h (**b**), and 24 h (**c**). The concentration of inflammatory cytokines, including PGE2, IL-8, gradually increased in a time-dependent manner except in TNF-α (**d**) (*P < 0.05, *n* = 5). *Scale bar* 500 nm

Representative TEM micrographs showed bacterial phagocytosis (Fig. 6a–c) at 2, 4, and 24 h, respectively. The rod shape of *Pg* strain W83 can be seen in the micrographs (arrow, Fig. 6a–c). All bacteria activated a variety of inflammatory cytokines, including PGE2, IL-8, and TNF-α, which gradually increased in a time-dependent manners. Interestingly, only interaction of TNF-α was then continuously decrease after reach to the highest point at 4 h. For PGE2 and TNF-α, the detection was highest at 24 and 4 h, respectively, after bacterial inoculations when compared to other time intervals (*P* < 0.05) (Fig. 6d).

### Karyotype analysis of hEPLCs

**Fig. 7:**
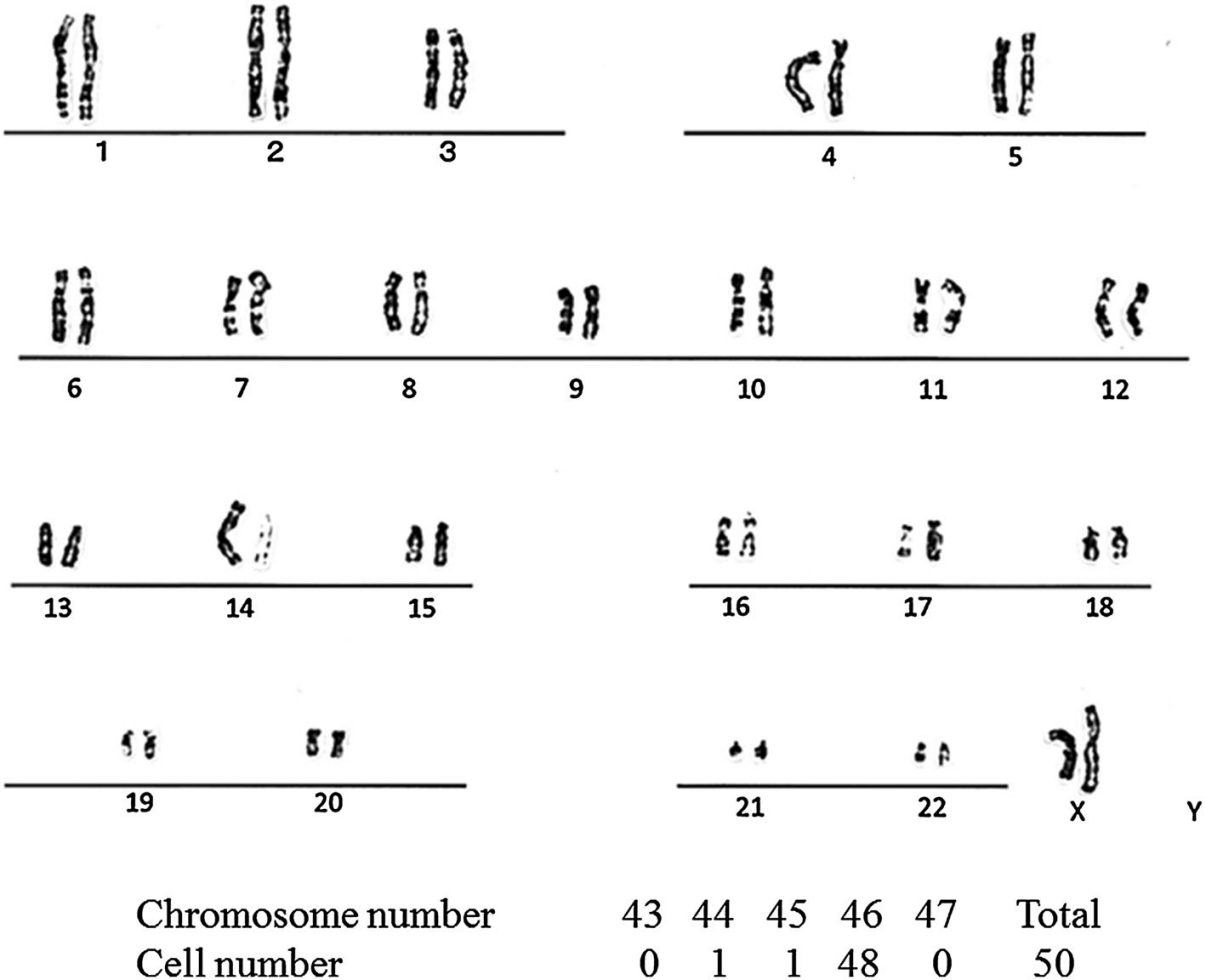
Distribution of the chromosome numbers in a representative hEPLCs line demonstrating a normal karyotype with diploid sets of chromosomes (2n = 46, XX)

To determine the chromosomal stability of the hEPLCs cell lines, we performed a G-banded analysis of the karyotype. The hEPLCs exhibited a normal karyotype with diploid chromosome number (2n = 46, Fig. 7).

## Discussion

The periodontal ligament is a specialized tissue involved in regeneration, wound healing, cell differentiation, and inflammation. Although periodontal disease is an inflammatory disease with a high incidence worldwide, the cell functions, inflammatory pathways, cell–cell interactions, and signal transductions of the PDL remain unclear.

This study investigating novel hEPLCs lines revealed epithelial-like cell morphology, which was quite similar to that of ERM. However, their characteristics such as mRNA expressions, immunofluorescences, and TEER results have demonstrated several contradictions to ERM. For cellular morphologies, hEPLCs and ERM were highly orientated and presented in an evenly small, pavement-like arrangement, but after prolonged culturing over 2 weeks, hEPLCs provided tight multilayers in spherical colonies differently from ERM. Previous studies established that ERM appeared to show polyhedral-shaped features in monolayered with high nuclear-cytoplasmic ratio [19–21]. Moreover, we identified hEPLCs that demonstrated in epithelial-like cell in cellular morphology, but their organelles showed secretory cell characteristics, which could be depicted in coated vesicle in TEM.

Herein, we proposed that the possibilities of hEPLCs derived from three possible origins. First, hEPLCs might derive from differentiated ERM, undergoing stem cell characteristics. The ERM derived from HR is particular remained in periodontal ligament tissue after complete root formation, but their existent and functions remain unclear. However, ERM cells are terminally differentiated epithelial cells that derive from an ectomesenchymal lineage. Nevertheless, previous studies confirmed that ERM cells contain unique stem cells capable of trans-differentiating between ectodermal and mesenchymal lineages, which retained their migratory state under suite conditions that drive osteogenic differentiation [12], demonstrating multilineage differentiation potentials in osteogenic, adipogenic, chondrogenic, and neurogenic lineage both in vitro and in vivo [11, 12]. Therefore, ERM cells might have the potential to provide high activity during trans-differentiation to PDL tissues [22, 23] and hEPLCs might possibly be the differentiated ERM that undergone from stem cells population. When heterogeneously existing among specific environment in periodontal ligament tissues, complex cell interactions with an abundant of signaling effects beyond their environment might induce unique target cell functions, and their characteristics of ERM such as self-renewal, proliferation and differentiation properties for capable to differentiate into the hEPLCs. Moreover, even the ERM-related cellular activities in periodontal regeneration and cementum differentiation have not yet been completely determined, and our results support the idea that hEPLCs are an active cell type that play a pivotal role in the PDL attachment apparatus as demonstrated by the cell aggregation from TEER results and in TJ protein expression imitating ERM. However, hEPLCs results in immunofluorescence of amelogenin and ameloblastin were negatively shown providing that hEPLCs-derived differentiated ERM might intervene within the PDL environment.

Secondly, hEPLCs might be derived from junctional epithelium (JE) of the dento-gingival unit in the periodontium. JE is a unique structure characterized by non-keratinized stratified squamous epithelium located underneath at the cementoenamel junction in healthy tissue [24], and exhibiting in long JE that can be penetrated to the cervical or middle one-third of the root surface after periodontal wound repairing. Particular behaviors of JE contribute to attenuating periodontal bacteria, forming epithelial barriers, and playing the pivotal role of cell adhesion. Previous studies have shown that basal lamina of JE similar to epithelial and endothelial cells in its laminin content, indicating that JE is involved in cell adhesion mechanism [25, 26]. Moreover, JE is originally derived from reduced enamel epithelium, by which transformed reduced ameloblasts change their morphology and function from short columnar secretory epithelial cell to flatted stratified squamous cells [27]. Our results of hEPLCs exhibited some characteristics similar to JE, including secretory function founding coated vesicles. Moreover, hEPLCs appeared to demonstrate host-protective mechanism against *Pg* and *Aa* invasion. The cells rapidly responded to the bacteria within 24 h, indicating hEPLCs might provide the host defense mechanism. We detected the time-dependent release of several cytokines, PGE-2, IL-8, and TNF-α, after inoculation. The results confirmed that hEPLCs phagocytosed the bacteria and appeared in elongated pseudopodia-like structure, covering rod-shaped bacteria. Thus, hEPLCs might be have a defensive bacteria mechanism similar to the JE.

Thirdly, the hEPLCs might be the cells that commit to trans-differentiate and reprogram by cellular switching among periodontal ligament tissue environment via epithelial to endothelial to mesenchymal phenotype, as termed “epithelial to mesenchymal transition” (EMT), “endothelial to mesenchymal transition” (EndMT), and “mesenchymal to epithelial transition” (MET) in diversity of biologic organism formations [11, 28–30]. According to characteristics of hEPLCs, they incorporated with all epithelial, endothelial, and mesenchymal features. They positively stained with CK-14, which is the odontogenic epithelium located in almost all cells of enamel organ and strongly expressed in the basal layer of gingival epithelium, JE and ERM. Moreover, TJ protein and their mRNA expressions were strongly demonstrated in Claudin-1-3, ZO-1, and OCLN, which are major markers dominating the paracellular of cell–cell adhesion in both epithelial and endothelial cells. While for mesenchymal lineage, mitochondria and vimentin expression showed in hEPLCs of immunofluorescence results. The EMT, EndMT, and MET are cellular processes that can switch phenotype among those three cell types when encompassing proper signaling stimulations, whether in development through pathologic circumstance such as malignant cellular transformation. To address EMT process in odontogenic structure, Akimoto et al. [23] indicated that HR, which are surrounded by mesenchyme, were partially merged phenotypic of both epithelial and mesenchymal cells expressing CK-14 and vimentin via transforming growth factor-β (TGF-β) stimulation following upregulation in snail 1 and 2 gene expression. The signaling pathway of TGF-β superfamily was mainly proposed as the most significant signaling pathway for EMT, EndMT, and MET [28, 31, 32]. These signaling pathways were not only potentially interplayed in odontogenic development, but also examined in other somatic organs such heart, kidney, liver, or skin [28]. Therefore, hEPLCs might be the cells that committed in this complex EMT, EndMT, and MET cascades due to neural crest origins, which can express both characteristics of ectodermal and mesodermal lineage, and thus hEPLCs might exhibit characteristics of cells differentiating across lineages.

Evidence of OCLN TJ marker in ERM negatively expressed suggesting that ERM lines were established from early stage of odontogenesis. OCLN is a cellular protein at a later stage of odontogenesis, indicating weak staining in pre-ameloblasts, but stronger staining in differentiated and secretory ameloblast [33] as vigorously shown in hEPLCs. Moreover, the OCLN protein has coiled-coil domains of COOH-terminal fusion protein, specifically bound to ZO-1 and most likely modified the Claudin-based properties to regulate mature TJ strands of the paracellular area [34, 35]. Thus, the interactions of OCLN with ZO-1 and Claudin suggested that hEPLCs aggregations have a meaningful cellular functions such as ion transportation and defense mechanism from bacterial infection, as shown in the high expression in all TJ markers proteins. These characteristics are similar to those of endothelial cells of the blood–brain barrier (BBB), which is interconnected by the TJ [36–38]. TJ in the BBB typically provide high TEER and demonstrate the important role of paracellular diffusion and protein transportation [39–41]. Our results indicated that hEPLCs developed high TEER, as similar as transmembrane protein in TJ and co-expressed in ZO-1, Claudins, and OCLN, which have an evidence that ZO-1 capable bind to Claudins directly and OCLN via the Guk domain [42, 43]. Therefore, cellular aggregation of hEPLCs might help to maintain the selected permeability between interacting cells, including ion, water, or protein exchange, and might act as functional barriers for pathogenic bacteria as resembling the BBB [44–46].

Therefore, we concluded that the hEPLCs were novel cell lines exhibiting uncommitted differentiated functions derived from ectomesenchymal characteristics [47]. They showed complex characteristics and specialized functions, creating the selective substance barrier in PDL tissue and providing pathologic defense mechanisms. The hEPLCs might provide strong protection inside PDL tissue by TJ-mediated cell–cell adhesion as similar to general epithelial and endothelial cells. Moreover, they demonstrated merged characteristics of all epithelial, endothelial, and mesenchymal cells, which might switch the trans-differentiation among complex environment. However, hEPLCs likewise derived from stem cells of ERM. Therefore, further in vivo studies and several functional analyses should be performed to investigate the specific locations of these cells and their specialized functions.

## Electronic supplementary material

Below is the link to the electronic supplementary material.

**Supplementary Figure 1.** (A) hEPLCs had an oval shape on day 1. (B) hEPLCs on day 3 exhibited extended processes to form a polyhedral morphology. (C) On day 7, the cell colonies developed into clusters with less intercellular space. (D) Around day 10, the hEPLCs developed a high nuclear to cytoplasmic ratio. (E) From 2 weeks to 3 weeks (F), the hEPLCs colonies were dense and formed multilayers. *Scale bar* of A-E = 50 µm. *Scale bar* of F = 100 µm (TIFF 864 kb)

Supplementary material 2 (TIFF 139 kb)
