# Supplementary figures and images for: Establishment and characterization of novel epithelial-like cell lines derived from human periodontal ligament tissue in vitro
Source: Hum Cell. 2017 Apr 22;30(4):237–48. doi: 10.1007/s13577-017-0173-y (PMC5646140; doi:10.1007/s13577-017-0173-y)

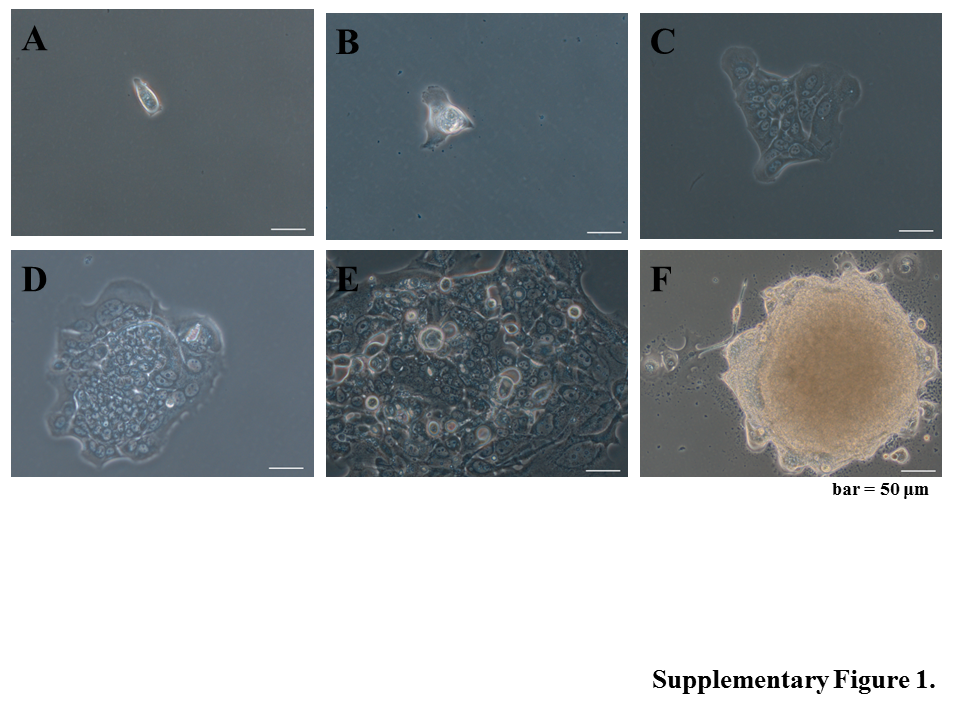

Supplement: Supplementary file 1 — Supplementary Figure 1. (A) hEPLCs had an oval shape on day 1. (B) hEPLCs on day 3 exhibited extended processes to form a polyhedral morphology. (C) On day 7, the cell colonies developed into clusters with less intercellular space. (D) Around day 10, the hEPLCs developed a high nuclear to cytoplasmic ratio. (E) From 2 weeks to 3 weeks (F), the hEPLCs colonies were dense and formed multilayers. Scale bar of A-E = 50 µm. Scale bar of F = 100 µm (TIFF 864 kb) [file 13577_2017_173_MOESM1_ESM.tiff]

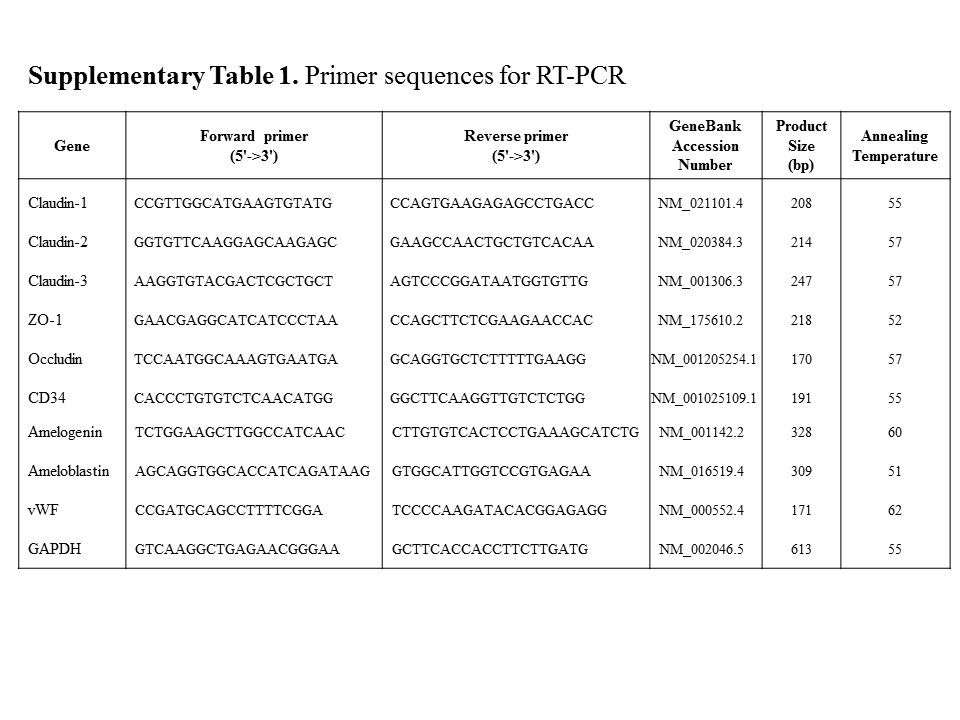

Supplement: Supplementary file 2 — Supplementary material 2 (TIFF 139 kb) [file 13577_2017_173_MOESM2_ESM.tiff]
